# Respiratory symptoms, sensitisation and occupational exposure in the shrimp processing industry

**DOI:** 10.3389/falgy.2025.1520576

**Published:** 2025-03-20

**Authors:** Fikirte Debebe Zegeye, Pål Graff, Miriam Grgic, Steen Mollerup, Anani Komlavi Afanou, Berit Elisabeth Bang, Karl-Christian Nordby, Anne Straumfors, Johanna Samulin Erdem

**Affiliations:** ^1^ Department of Occupational Toxicology, National Institute of Occupational Health, Oslo, Norway; ^2^ Faculty of Medicine, University of Oslo, Oslo, Norway; ^3^ Department of Occupational Chemistry, National Institute of Occupational Health, Oslo, Norway; ^4^ Department of Occupational and Environmental Medicine, University Hospital of North Norway, Tromsø, Norway; ^5^ Department of Medical Biology, Faculty of Health Sciences, UiT, The Arctic University of Tromsø, Tromsø, Norway; ^6^ Department of Occupational Medicine and Epidemiology, National Institute of Occupational Health, Oslo, Norway

**Keywords:** allergy, respiratory symptoms, biomarkers, irritants, sensitisation, tropomyosin

## Abstract

**Introduction:**

Shellfish processing workers are highly susceptible to respiratory illnesses such as allergies and asthma. This study examined respiratory symptoms and biomarkers of allergy and asthma in Norwegian shrimp processing plant workers and evaluated allergenic and irritant protein exposures in the workplace.

**Material and methods:**

The study included 35 shrimp processing workers and 21 controls. Respiratory symptoms were assessed via questionnaire; blood samples were analysed for allergy and asthma biomarkers and specific IgE levels. Air samples were analysed for protein levels and composition.

**Results:**

Shrimp processing workers had four to five times higher odds of reporting acute upper and chronic lower respiratory symptoms than the controls. They also had significantly higher plasma levels of IL4, CCL20, CSF2 and MMP12, with 11% of the exposed workers showing elevated levels of shrimp and crab specific IgE. Furthermore, exposed workers showed increased plasma levels of SFTPD and CHI3L1 post-shift. The median total protein exposure was 6 µg/m^3^, with peaks up to 66 µg/m^3^ in the cooking and peeling department. Total protein levels were correlated with CCL20, IL13, and basophil counts. Ninety-five shrimp proteins were identified, including seven known and eight potential allergens. Tropomyosin levels were generally high, particularly in the cooking and peeling department.

**Conclusion:**

Shrimp workers had a higher prevalence of respiratory symptoms and biomarkers of allergy and asthma. The work environment contained tropomyosin and other allergenic proteins as well as irritants, highlighting the need for protective measures, especially in the cooking and peeling departments.

## Introduction

The seafood industry constitutes a large part of the Norwegian food industry, and seafood production has hit a record export volume of 2.9 million tons of seafood to a value of (€13,4 bn/£11.8 bn) in 2022 (Norwegian Seafood Council).

Globally, over 61 million individuals work in the seafood industry, and respiratory health effects such as allergies and asthma are notably prevalent among these workers (1–3). The prevalence of occupational rhinitis and occupational asthma among these workers ranges from 2% to 36% (2, 4, 5). Workers in the shellfish industry have a higher frequency and severity of respiratory symptoms and are more often sensitised than those in the fish industry (2, 6).

The seafood industry is characterised by complex exposure to chemicals and biological factors (bioaerosols). These bioaerosols include allergens, proteases, microorganisms and endotoxins (6–11). Bioaerosol exposures occur primarily through inhalation of dust, steam, vapour, and aerosolised proteins generated during butchering, thawing, cooking or boiling, peeling, and grinding (12, 13). Thermal processes such as thawing and boiling can potentially accelerate the evaporating process, leading to the formation of exposure hot spots for allergenic proteins with enhanced allergenicity (13).

Tropomyosin and arginine kinase are the two most extensively studied allergenic proteins in shellfish (14, 15). However, over the past decade, several additional allergens have been identified, including sarcoplasmic calcium-binding, myosin light chain, hemocyanin, pyruvate kinase, and enolase (7, 14–18). Exposure to these proteins may lead to occupational rhinitis and asthma, commonly through IgE-mediated allergic reactions (16, 18). Moreover, chitin, which is a biopolymer of N-acetyl-*b*-D-glucosamine, in the exoskeleton of shellfish, insects, and fungi has been shown to induce inflammation through the production of reactive oxygen species (ROS) in humans, and induction of chitinase in the murine lung that could lead to the development of bronchial hyperreactivity in an IgE independent manner (19–21). Further, proteases such as trypsin may trigger inflammation, respiratory tract hyperresponsiveness and possibly IgE-mediated sensitisation upon inhalation (22–24). Moreover, king crab and salmon trypsin are potent stimulators of protease-activated receptors causing pro-inflammatory responses in pulmonary epithelial cell models (23, 25–27).

Exposure to bioaerosols can trigger hypersensitivity reactions in the respiratory tract, potentially leading to decreased lung function, occupational asthma, and occupational rhinitis (28, 29). An immunologically mediated sensitisation primarily induces occupational asthma, but non-immunologic and irritant-induced cases have also been reported (28, 30). In immunologically mediated sensitisation, several chemokines and cytokines are involved, with the top three important cytokines being interleukin 4 (IL4), IL5 and IL13 (31, 32).

To date, studies of occupational exposure and respiratory effects in shrimp processing plants are scarce. However, among crustaceans, shrimp is the most common elicitor of allergic reactions. The northern shrimp (*Pandalus borealis*) is one of the least studied in terms of allergenic protein profiles and health impacts associated with occupational exposure during processing. Therefore, this study aimed to evaluate the prevalence of respiratory symptoms in two Norwegian shrimp processing plants and assess specific IgE and biomarker levels in the blood of exposed workers. Additionally, the study sought to characterise the protein composition of air samples collected from different work processes in the processing plants, with a focus on identifying allergenic proteins and assessing potential respiratory health effects associated with these proteins.

## Materials and methods

### Study design and study population

A cross-sectional study was conducted on workers in two Norwegian shrimp processing plants. The processing plants operate two to three shifts per day, five days a week. The inclusion criteria for the study were to be employed at one of the two selected shrimp processing plants during the study period (May and June 2022) and age above 18 years. The study was approved by the Regional Committee for Medical and Health Research Ethics (384542).

A statistical power analysis was performed for a dichotomous endpoint, aiming for 80% power at a 0.05 significance level using an occupational asthma prevalence of 36% in the shellfish industry (for the exposed group) and a current asthma prevalence of 5.4% in the general population (for unexposed control) (2, 33). The analysis concluded that the study should include at least 35 exposed workers and 18 unexposed control workers. Accordingly, all exposed workers in the shrimp processing plants (*n* = 65) were invited with a letter informing them about the study and its objectives. Of these, a total of 35 exposed production workers in shrimp processing plants participated in one or more parts of the study. Anonymised data from 21 administrative personnel in waste sorting plants were included as controls under ethical approval number 34312. These workers shared a similar sociodemographic background and had no prior experience in the seafood industry. Written informed consent for questionnaires and blood sampling was obtained from the study participants. Blood samples were collected from the exposed workers at two different times, pre-shift and post-shift, while a single sample was taken from the control workers. A total of 30 personal air samples and 2 stationary air samples were collected.

### Questionnaire

All employees were asked to complete a questionnaire that gathered personal information, including age, sex, height, and weight. Additionally, it inquired about their smoking or snuffing habits and whether they had been diagnosed with asthma, allergy, eczema, or experienced acute and chronic respiratory symptoms (Supplementary Data 1).

### Blood sampling and analysis

A total of 28 exposed workers and 21 control workers consented to give blood samples. Of the exposed workers, 25 workers supplied two blood samples, one before and one after shift, to examine acute health effects. The remaining exposed workers were unwilling or unable to provide blood samples after the shift. All the control workers provided one blood sample. Blood samples were collected using EDTA (BD Vacutainer K2E, BD, USA) and serum tubes (BD Vacutainer SST II Advance, BD, USA). Following the blood collection, the EDTA tubes were inverted 8–10 times and centrifuged for 10 min at 2,100 × g to separate plasma from blood cells, while the serum tubes were left to coagulate for 30 min at room temperature and then centrifuged at 2,100 × g for 5 min. Plasma samples were aliquoted and stored at – 80°C until further analysis. The whole blood and serum were sent to an accredited laboratory, Fürst Medicine Laboratory (Oslo, Norway), for differential counts of white blood cells (WBC) and analysis of specific IgE antibodies against salmon, crab, and shrimp, using the sandwich immune assay ImmunoCAP^TM^ (Thermo Fisher Scientific, Waltham, Massachusetts, USA). The three species were selected due to their potential cross-reactivity. While fish allergens typically do not cross-react with shellfish, a previous study showed that tilapia's tropomyosin allergen shared 53.5% homology with shrimp tropomyosin (34). The standard clinical reference range for WBC analysis were total leukocytes (3.5–10 × 10^9^/L), neutrophils (1.5–7.3 × 10^9^/L), lymphocytes (1.1–3.3 × 10^9^/L), monocytes (0.2–0. 8 × 10^9^/L), eosinophil (<0.4 × 10^9^/L) and basophil (<0.2 × 10^9^/L). The normal reference range for the IgE levels was <0.35 KU/L. Workers who had values exceeding these ranges were reported in percentages.

### Biomarker analysis

Biomarkers of allergy and asthma were measured in plasma samples using a custom multiplex human Luminex discovery assay (R&D Systems, Minneapolis, MN, USA). The custom multiplex assay included C-C motif chemokine ligand 17 (CCL17), CCL20, CCL26, IL4, IL5, IL13, IL33, chitinase 3-like 1 (CHI3L1), matrix metalloproteinase 12 (MMP12), periostin (POSTN), Surfactant Protein - D (SFTPD), thymic stromal lymphopoietin (TSLP) and colony-stimulating factor 2 (CSF2) with a sensitivity ranging from 0.5 to 95.7 pg/ml for the different biomarkers. These biomarkers were selected based on their key roles in the Th2 immune response, crucial in asthma and allergy pathophysiology. Chemokines CCL17, CCL20, and CCL26 attract immune cells to inflamed sites, enhancing allergenic reactions (35, 36). Cytokines IL4, IL5, IL13, and IL33 support IgE production, eosinophil activation, and airway hyperresponsiveness (31, 32, 37, 38). CHI3L1 and MMP12 mediate airway tissue remodelling and inflammation (39–41). While POSTN exacerbates inflammation by regulating cellular interactions and supporting tissue integrity, SFTPD protects lung tissue by modulating immune responses (42, 43). TSLP initiates allergic reactions through dendritic cell activation, and CSF2 regulates inflammation by promoting granulocyte and macrophage production (32, 35, 37, 38).

The plasma samples were diluted 1:2 and analysed according to the manufacturer's instructions. All samples were analysed in parallel using the xMAP INTELLIFELEX instrument (R&D Systems). Biomarker concentrations in the plasma samples were estimated using a six-point standard curve with the Quantist data analysis software version 1.0.1.0 (R&D Systems). IL33 and TSLP levels were below the limit of detection (LOD) and were consequently excluded from further analysis.

### Aerosol sampling

Active air sampling was performed using a Conical Inhalable Sampler (CIS) (Casella Solutions, Kempston, UK) attached to Casella Apex2 pumps (Casella UK, Wolseley Rd, Kempston, Bedford, UK) over two consecutive days. The sampler was loaded with a 37 mm polytetrafluorethylene (PTFE) filter (1.0 µm, Millipore, Billerica, MA, USA). The airflow was set to 3.5 L/min following calibration with a digital flow meter Defender 520 M (Defender; SKC Inc., Eighty-Four, PA, USA).

Workers who consented to participate in the personal air sampling campaign carried the samplers in a small backpack, positioning the sampling cassettes in their personal breathing zone for the entire 8–9 h work shift. Sampling was done across five different work processes: (1) Thawing—thawing frozen shrimp blocks (2) Truck Driving—transporting frozen shrimp to thawing, maturation and subsequently to the cooking area. Thawers and truck drivers work in the same department but perform different tasks. (3) Technician—operating machinery for cooking and peeling the shrimp (4) Packing—conducting quality control and performing automated packing of shrimp, and (5) Flour production—drying and grinding shrimp shells to produce flour.

In addition to the personal air samplers, one stationary sampler was included in the cooking and peeling area per processing plant. The stationary samplers were equipped with PTFE filters and were placed in the cooking and peeling department. Sampling was performed for 11–23 h.

### Protein extraction from PTFE filters

Proteins were extracted from the personal PTFE filters with 0.5% PBS-Tween 20 buffer. The samples were rotated for 4 h at 4℃ followed by centrifugation at 1,200 × g for 10 min. The supernatants were aliquoted and stored at −80℃ until further analyses of total protein, proteolytic activity, and measurements of allergen levels. Stationary samples for LC-MS/MS analysis were extracted using a 10 mM glycine/0.5% SDS buffer (pH 7.55). The samples were rotated for 16 h at 4℃ followed by centrifugation at 1,200 ×  g for 10 min. Supernatants were stored at −80℃ until proteomic analysis. The total protein levels were quantified using QuantiPro BCA Assay Kit (Sigma-Aldrich, St. Louis, USA) and measured with a Multiscan FC (Thermo Scientific) spectrophotometer at 560 nm according to the manufacturer's instructions.

### Proteolytic activity by zymography

Proteolytic activity was assessed using zymography, a sodium dodecyl sulfate-polyacrylamide electrophoresis impregnated with 0.1% gelatin substrate in a polyacrylamide gel. The gelatine degrades in the presence of protease, creating unstained regions on the gel as previously described (9). The protease activity was calculated based on a five-point standard curve (0.1–0.9 nM) from porcine trypsin with known enzyme activity. The gel image was captured using the BioDoc® Imaging system. The intensity of the degraded regions (unstained regions) was quantified using image analysis software (UVP Vision Works®, Analytik Jena GmbH + Co).

### Proteomic analysis by LC-MS

LC-MS analysis was performed on stationary samples collected in the cooking and peeling department to assess the protein composition of the bioaerosol samples. The analysis was conducted at the Proteomic and Metabolomics Core Facility (PRiME) at the Arctic University of Norway (UiT). The filter extract eluate was evaporated to dryness and resuspended in 2% SDS 50 mM TEA buffer and sonicated for 3 min in a water bath. Protein digestion was done following the S-trap micro protocol (protifi.com). The eluted peptide solution underwent evaporation and was subsequently reconstituted in 0.1% formic acid. The samples were analysed on an Easy nLC 1,200 instrument coupled to an Orbitrap Exploris 480 for LC-MS analysis.

Protein sequences were blasted in the National Center for Biotechnology Information (NCBI) protein BLAST database for identification. Only 85 proteins are available from the species *Pandalus borealis* in the reference database. Therefore, most identification was based on homology in the infraorder Caridea. A mass spectrometry contaminant database (MaConDa) was used to remove contaminant proteins from the samples.

Protein allergenicity prediction was performed using the AllerCatPro2.0 tool on proteins that had three or more peptides match (44). The tool identifies proteins with potential allergenicity by analysing their amino acid sequences and predicted 3D structure, comparing them against comprehensive allergen databases, and categorising them as strong, weak or no evidence for allergenicity.

### Measurements of tropomyosin and arginine kinase levels

Tropomyosin levels in the personal air samples were measured using a shrimp tropomyosin ELISA kit (Inbio, Charlottesville, VA, USA) with a 0.2 ng/ml limit of detection, according to the manufacturer's instructions. The tropomyosin concentration was determined using a 10-point standard curve (0.1–50 ng/ml). The absorbance was measured at 450 nm using Bio Tek Synergy Neo2 Hybrid Multimode Reader (Agilent Technologies, USA).

Arginine kinase was analysed by western blotting. Due to the low protein concentration, the samples for analysis of arginine kinase were pooled per work process. Concentrated protein extracts were separated by Any kD MP Mini protein TGX stain-free protein gradient (Biorad, Hercules, CA) SDS-PAGE and transferred to a Trans-Blot turbo PVDF membrane (Biorad). The membranes were incubated with Pierce™ protein-free blocking buffer (Thermo Fisher Scientific) for 1 h at room temperature to prevent non-specific background binding. The membranes were incubated overnight at 4°C with primary antibody against arginine kinase (Abcam, Cambridge, UK) at a concentration of 3.5 µg/ml. After washing three times with Tris-buffered saline with 0.05% Tween-20 (TBST), the membranes were incubated with a secondary antibody, HRP-conjugated anti-rabbit IgG secondary antibody (Cell Signalling Technology, MA, USA), for 1 h at room temperature. After incubation, the membranes were washed three times with TBST and incubated with Super signal west Atto ultimate sensitivity substrate (Thermo Fisher Scientific) for 5 min. Signals were captured using a CCD camera-based imager (Amersham Imager 600, UK). Densitometric analysis was performed by ImageJ 1.54 g, and the obtained values were adjusted for the total air volume per filter.

### Statistical analysis

Statistical analysis was conducted using GraphPad Prism version 9 and R version 4.3.1. Power analysis was done using the pwr package by employing the pwr.2p2n.test function*.* Data sets that were not normally distributed, as determined by the Shapiro–Wilk test, or contained outliers identified by the Q-Q plot were log-transformed before analysis. This was followed by a sensitivity analysis when necessary. The log transformation specifics and analysis types are indicated in the footnotes of each result. Variations in age, years of employment, and body mass index (BMI) between groups were evaluated using an independent sample *t*-test. Differences in sex, smoking status and symptom prevalence between groups were assessed using Pearson Chi-square or Fisher's exact test. Multivariate logistic regression was used to evaluate the association between exposure status and the odds of reporting respiratory symptoms, adjusting for potential confounding variables. Multivariate linear regression was employed to examine the relationship between biomarker levels and exposure status, adjusting for potential confounding variables. The confounding variables were selected for both models based on their influence on the model using stepwise selection. The initial regression model included all potential confounders: age, sex, smoking status, BMI, length of employment, asthma, and allergy. Only variables that significantly impacted the dependent variable were retained, while others were excluded. Additionally, we assessed the associations between biomarker levels and acute and chronic respiratory symptoms using linear regression while accounting for potential confounding effects. *p* < 0.05 was considered significant. A paired sample *t*-test or Wilcoxon signed-rank test was used to compare biomarker levels in exposed production workers' blood before and after work. Comparisons of exposure levels between different workgroups were done using one-way ANOVA or the Kruskal–Wallis test. Pearson correlation was used to assess the correlation between biomarker levels, differential counts of WBC, total protein and previously reported endotoxin levels.^1^

## Results

### Study population characteristics

The study population consisted of 56 workers, of whom 43 were men and 13 were women. Their ages ranged from 20 to 65, with a mean BMI of 26–28 (Table 1). Current and past smoking habits of the exposed workers and controls were similar. The comparison of the sociodemographic variables revealed no significant difference between the two groups (Table 1).

**Table 1:** Characteristics of the study population.

Exposure status	Number (*N* = 56)	Mean age yrs. (SD)^a^	Sex male/female %^b^	Mean BMI (SD)^a^	Current smokers %^b^	Ex smokers %^b^	Mean employment yrs. (SD)^a^
Exposed workers	35	39 (14)	77/23	28 (14)	11.4	47.6	8.2 (14)
Controls (unexposed workers)	21	43 (8)	76/24	26 (8)	9.5	42.9	5 (8)

^a^ Independent sample *t*-test was used, no significant difference was observed.

^b^ Fisher test was used, no significant difference was observed.

### Prevalence of self-reported respiratory problems

The prevalence of reported doctor-diagnosed asthma was more frequent in exposed workers (22.9%) than in controls (4.8%), but the difference was not statistically significant (Supplementary Table S1). In the past year, 2.8% of the exposed workers reported experiencing asthma attacks, and 5.7% reported using asthma medication, while no asthma attack or medicine use was reported among the control group. 11.4% of exposed workers and 4.7% of control were diagnosed with asthma as adults (Supplementary Table S1). Self-reported allergy was more frequent among controls (42.9%) than exposed workers (26.2%) (Supplementary Table S1). However, this was not statistically significant, and the reported allergies were predominantly grass or pollen allergies in both groups. In addition, 2.9% of exposed workers reported shrimp allergy (Supplementary Table S1).

All individual respiratory symptoms were relatively more frequent in exposed workers, ranging from 8.5% to 45.7%, compared to controls (0%–19%) (Table 2). Chronic lower and acute upper respiratory symptoms were significantly prevalent among the exposed workers compared to the controls (*p* = 0.02) (Table 2). Furthermore, the odds of having chronic lower respiratory symptoms were four times higher in exposed workers than in controls (*p* = 0.01) (Table 3). In addition, exposed workers had a five times higher likelihood of reporting acute upper respiratory symptoms than controls, with a significant *p*-value of 0.01 in a logistic model adjusted for smoking (Table 3). Although it was not statistically significant, acute lower respiratory symptoms were more than twice as frequent among exposed (37.1%) than control (14.3%) (Table 2).

**Table 2:** Prevalence of self-reported respiratory symptoms.

No.	Respiratory symptom^a^	Exposed workers	Controls (unexposed workers)
		*N* = 35	*N* = 21
1	Regular trouble breathing (%)	31.4	9.5
2	Shortness of breath (%)	11.4	4.7
3	Wheezing in the chest (%)	28.6	14.3
4	Produce phlegm in the morning (%)	8.5	0
5	Cough daily for more than 3 months (%)	17.1	4.7
	Chronic lower respiratory symptoms (1–5) (%)^b^	**62**.9*	**28**.**6**
6	Sneezing (%)	34.3	19
7	Stuffy nose (%)	45.7	9.5
8	Runny nose (%)	34.3	19
9	Sore throat (%)	28.6	14.3
	Acute upper respiratory symptoms (6–9) (%)^b^	**68**.6*	**33**.**3**
10	Cough with phlegm (%)	28.6	0
11	Cough with sputum (%)	14.3	9.5
12	Wheezing in the chest (%)	14.3	4.7
13	Congested chest (%)	14.3	4.7
	Acute lower respiratory symptoms (10–13) (%)^b^	**37**.**1**	**14**.**3**

^a^ Workers were categorised as having acute and or chronic respiratory symptoms if they reported experiencing any of the symptoms described in the same category.

^b^ Pearson chi-square test was used.

* Significant difference from controls, *p* = 0.02.

**Table 3:** Association between exposure status and respiratory symptoms.

Respiratory symptoms^a^	Odds ratio (OR)^b^	95% confidence interval (CI)^b^	*p*-value	Adjusted for
Chronic lower respiratory symptoms	4.0	1.2–13.9	0.01	–
Acute upper respiratory symptoms	5.0	1.4–19.4	0.01	Smoking
Acute lower respiratory symptoms	2.3	0.6–13.4	0.2	Smoking & length of employment

^a^ The initial model included age, sex, smoking status, BMI, length of employment, asthma, and allergy as confounders and covariates. Influential predictors were adjusted after stepwise selection. The variables adjusted for each model are shown in the “Adjusted for” column.

^b^ The OR and CI are derived from the logistic regression model.

### Specific IgE test and differential leukocyte count

Elevated specific IgE levels against crab and shrimp allergens were detected in 11% of the exposed workers. An additional 3.7% of the exposed workers had elevated IgE against salmon (Supplementary Table S2). Furthermore, 7.1% of the exposed workers had elevated total leukocytes, lymphocytes, monocytes, and eosinophils. While the number of basophils was normal in all the production workers, 3.5% had neutrophil counts higher than the acceptable range (Supplementary Table S2).

### Biomarkers for allergy and asthma

Linear regression analysis revealed that, after adjusting for confounding factors, IL4, CCL20, MMP12 and CSF2 levels were significantly higher in exposed workers compared to the controls, whereas CCL17 levels were significantly lower than controls (Table 4). Being in the exposed group was associated with a higher level of IL4 with an estimate of 8.5 pg/ml (95% CI: 1.5–15.5), *p* = 0.02; CCL20 with an estimate of 2.3 pg/ml (95% CI: 1.3–4.3), *p* = 0.01; MMP12 with an estimate of 3.1 pg/ml (95% CI: 1.2–8.1), *p* = 0.02; and CSF2 with an estimate of 1.4 pg/ml (95% CI: 1.0–1.9), *p* = 0.03 (Table 4). On the contrary, being in the exposed group was associated with a lower level in CCL17 with an estimate of 0.7 pg/ml (95% CI: 0.6–0.9), *p* = 0.01 (Table 4). No effects were observed for IL5, IL13, CCL26, SFTPD, CHI3L1, and POSTN. Sex, age and self-reported allergy were identified as influential covariates for three or more biomarkers, whereas smoking status did not influence any of the biomarkers tested (Table 4). Asthma and allergies were significantly associated with CCL20 and CCL17, *p* < 0.03, respectively (Table 4). Raw data is available in the (Supplementary Data 2).

**Table 4:** Linear regression analysis of allergy and asthma biomarker levels in workers’ plasma.

Biomarkers^a^	Variables	Estimate/exp estimates (pg/ml) (95% CI)	*p*-value
IL4	(Intercept)	22.5 (16.4–28.6)	0.00
	Exposure status^c^	8.5 (1.5–15.5)	0.02
	Allergy	5.2 (−2.1–12.4)	0.16
IL5^b^	(Intercept)	1.7 (1.2–2.5)	0.01
	Exposure status^c^	1.1 (0.8–1.6)	0.41
	CLRS	1.3 (0.9–1.7)	0.16
IL13	(Intercept)	58 (27–123)	0.00
	Exposure status^c^	1.5 (0.6–4.2)	0.39
	CLRS	1.8 (0.7–4.9)	0.22
CCL17^b^	(Intercept)	159 (120–210)	0.00
	Exposure status^c^	0.7 (0.6–0.9)	0.01
	Allergy	0.6 (0.5–0.8)	0.00
	CLRS	1.7 (1.3–2.2)	0.00
	ALRS	0.6 (0.5–0.8)	0.00
CCL20^b^	(Intercept)	66 (14–302)	0.00
	Exposure status^c^	2.3 (1.3–4.3)	0.01
	Asthma	2.7 (1.1–6.6)	0.03
	Allergy	0.6 (0.3–1.1)	0.11
	ALRS	0.5 (0.2–1.0)	0.05
CCL26^b^	(Intercept)	9.0 (8.2–9.9)	0.00
	Exposure status^c^	1.0 (0.9–1.1)	0.93
MMP12	(Intercept)	11.1 (5.5–22.8)	0.00
	Exposure status^c^	3.1 (1.2–8.1)	0.02
SFTPD^b^	(Intercept)	5,174 (2,629–10,184)	0.00
	Exposure status	1.32 (0.9–1.8)	0.08
	Allergy	1.4 (0.9–1.9)	0.06
CHI3L1^b^	(Intercept)	9,301 (4,549–19,017)	0.00
	Exposure status^c^	0.8 (0.6–1.2)	0.25
	CLRS	1.4 (1.0–2.1)	0.09
CSF2^b^	(Intercept)	0.8 (0.4–1.8)	0.59
	Exposure status^c^	1.4 (1.0–1.9)	0.03
	Allergy	0.8 (0.6–1.1)	0.17
	ALRS	0.7 (0.5–1.0)	0.07
POSTN^b^	(Intercept)	33,759 (24,798–45,958)	0.00
	Exposure status^c^	0.9 (0.8–1.1)	0.32

^a^ All-biomarker levels were log-transformed except for IL4; estimates or exponentiated (exp) estimates are presented. The initial linear regression model included age, sex, smoking status, BMI, length of employment, asthma, and allergy as confounders and covariates. Variables affecting the biomarker levels were selected via stepwise selection.

^b^ In addition to the variables shown in the table, IL5, CCL17, CCL20, and CCL26 were adjusted for sex; SFTPD, CHI3l and POSTN were adjusted for age; and CSF2 were adjusted for BMI and length of employment.

^c^ Exposure status: exposed workers vs. controls (reference), Sex: Male vs. female (reference), CLRS – Chronic lower respiratory symptoms, ALRS - Acute lower respiratory symptoms. Allergy, Asthma, CLRS and ALRS, yes vs. no (reference).

### Associations between reported respiratory effects and biomarker levels

Assessment of the associations between biomarker levels and respiratory symptoms showed that chronic lower respiratory symptoms were significantly associated with a higher level of CCL17 with an estimate of 1.7 pg/ml (95% CI: 1.3–2.2), *p* < 0.001. Acute lower respiratory symptoms were associated with lower CCL17 [estimate: 0.6 pg/ml, (95% CI: 0.5–0.8), *p* < 0.001] and a borderline significant lower level of CCL20 [estimate: 0.5 pg/ml (95% CI: 0.2–1.0), *p* = 0.05] (Table 4). No associations were found between acute upper respiratory symptoms and the analysed biomarkers.

A comparison of biomarker levels before and after work to assess the “acute effect” among exposed workers using a paired *t*-test showed a significant increase in the levels of SFTPD and CHI3L1, with a mean difference of 1.2 pg/ml (95% CI: 1.1–1.2), *p* < 0.001 and 1.0 pg/ml (95% CI: 1.0 to−1.1), *p* = 0.03 after the work shift, respectively. No effects were observed for the remaining biomarkers. Moreover, no differences were observed between workers performing different work tasks.

### Aerosol total protein concentrations and composition

**Figure 1:**
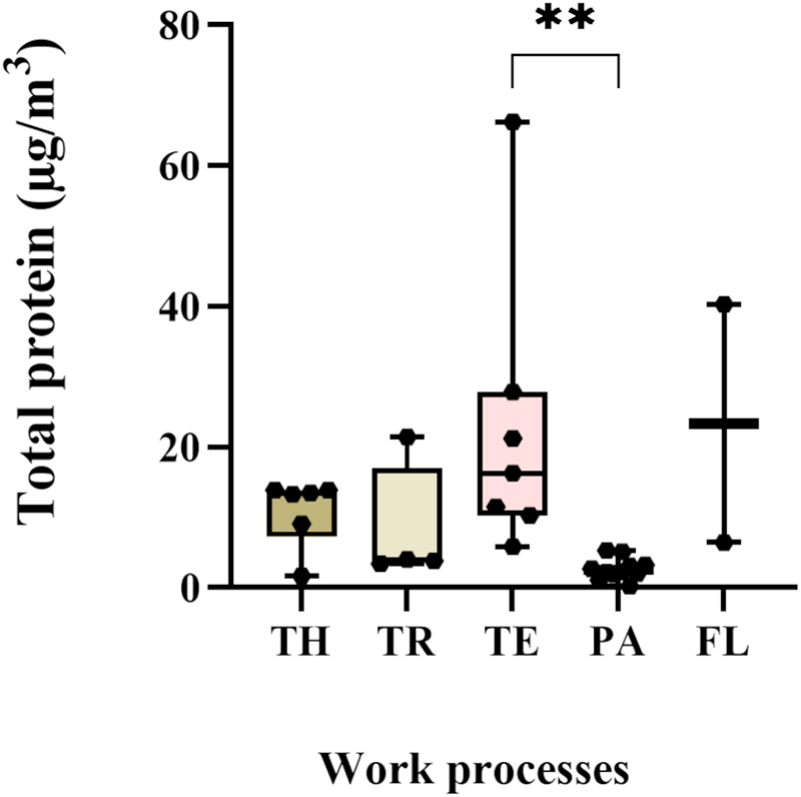
Exposure levels of total protein in personal samples. TH-thawers (*n* = 6), TR-truck drivers (*n* = 4), TE-technicians (*n* = 7), PA-packers (*n* = 11), and FL- flour production workers (*n* = 2). Group comparisons were done on log-transformed data using the Kruskal–Wallis test, followed by Dunn's multiple comparison test.

The total protein concentrations in the collected personal samples ranged from LOD to 66.2 µg/m^3^, with a median level of 6 µg/m^3^. The highest individual exposure was measured among the technicians, who also had overall higher protein exposure levels than the packers (*p* = 0.001) (Figure 1). Furthermore, the highest median exposure (23.4 µg/m^3^) was measured among flour production workers, while packers had the lowest median exposure (2 µg/m^3^) (Figure 1). Analyses of protein composition in the stationary collected aerosol samples resulted in the identification of 95 shrimp proteins. Of these, seven were known allergens: tropomyosin, arginine kinase, sarcoplasmic calcium-binding protein, troponin C, triosephosphate isomerase, myosin light chain and hemocyanin (Table 5). In addition, eight potential novel shrimp allergens were identified (Table 6). Of these, alpha-tubulin, heat shock protein 70, pyruvate kinase, troponin T, glyceraldehyde 3-phosphate dehydrogenase and myosin heavy chain showed strong evidence for allergenicity in the AllerCatPro2.0 prediction tool, whereas chitinase and vitellogenin demonstrated weak evidence of allergenicity. Detailed identification information is shown in Supplementary Table S3.

**Table 5:** Identified known shellfish allergens in stationary aerosol samples and their corresponding allergen codes.

Identified allergens			Previously characterised allergen codes			
Protein name	Accession #	Species	Homology between shrimp species	MW [kDa]	Allergen code	Ref.
Tropomyosin	ADC55380.1	*Macrobrachium rosenbergii*	99–100%	32.8	Cra c 1, Lit v 1, Pen b 1, Pen a 1, Pen i 1, Pen m 1, Met e 1	(15, 16, 45)
	BAF47264.1	*Pandalus eous*		32.7		
Arginine kinase	QQL13770.1	*Palaemon carinicauda*	>95%	39.5	Cra c 2, Lit v 2, Pen m 2, Mac r 2	(15, 16, 45)
	BAH56610.1	*Neocaridina denticulata*		39.5		
	QID75636.1	*Macrobrachium nipponense*		39.6		
	BAH56608.1	*Neocaridina denticulata*		39.5		
Sarcoplasmic calcium-binding protein	ACR43475.1	*Crangon crangon*	82–100%	22.1	Cra c 4, Lit v 4, Pen m 4	(15, 16, 45)
	ACR54113.1	*Palaemon varians*		7.7		
Troponin C	AQV08182.1	*Palaemon carinicauda*	81–100%	16.9	Cra c 6, Pen m 6	(15, 16, 45)
	ACR43478.1	*Crangon crangon*		16.8		
Triosephosphate isomerase	ACR43476.1	*Crangon crangon*	87–99%	27	Cra c 8, Pen m 8	(15, 16, 45)
Myosin light chain	ACR43477.1	*Crangon crangon*	86–87%	17.4	Cra c 5, Lit v 3, Pen m 3	(15, 16, 45)
	ACR54116.1	*Palaemon varians*	16–17%	17.3		
Hemocyanin	AEJ08191.1	*Palaemon carinicauda*	86–89.4%	76.5	Lit v HC, Pen m 7	(15, 16, 45)
	AVK43049.1	*Palaemon carinicauda*		78.4		
	ALN67306.1	*Macrobrachium rosenbergii*		76.5		
	AHJ90473.1	*Macrobrachium nipponense*		79.1		
	AEC46861.1	*Macrobrachium nipponense*		76.6		
	AJG06858.1	*Macrobrachium rosenbergii*		77.3		
	AJG06857.1	*Macrobrachium rosenbergii*		77.6		

**Table 6:** Novel allergens identified in stationary aerosol samples collected from the cooking and peeling department using AllerCatPro2.

Identified protein			Predicted most similar allergen		Similarity to allergen and resulting predicted evidence for allergenicity		
Protein name	MW [kDa]	Accession #	Protein allergen	Other species with similar allergen	% identity, linear 80 aa window	% identity, 3D epitope	Result
Alpha-tubulin	39.4	QBS13807.1	Lep d 33	*Lepidoglyphus destructor*	98.8	100	Strong evidence
Chitinase	54.2	AFC60658.1	Bla g 12	*Blattella germanica*	70	84.6	Weak evidence
Heat shock protein 70	71.2	ACL30943.1	Tyr p 28	*Tyrophagus putrescentiae*	95	100	Strong evidence
	66.3	ACL52279.1	Der p 28	*Dermatophagoides pteronyssinus*	92.5	100	Strong evidence
	71.5	ADN78256.1	Tyr p 28	*Tyrophagus putrescentiae*	98.8	100	Strong evidence
Pyruvate kinase	57.5	ALK82311.1	Sal s 9	*Salmo salar*	81.2	–	Strong evidence
Troponin T	45.7	AQV08184.2	Pon l 7	*Astacus leptodactylus*	93.8	–	Strong evidence
Vitellogenin	283.1	QCS40650.1	Der f 14	*Dermatophagoides farinae*	37.2	40	Weak evidence
	284.6	AHD26978.1	Der f 14	*Dermatophagoides farinae*	35	37.5	Weak evidence
	283	ACU51164.1	Der f 14	*Dermatophagoides farinae*	33.8	50	Weak evidence
	283.4	BAD11098.1	Der f 14	*Dermatophagoides farinae*	35	50	Weak evidence
	287.6	AFM82474.1	Der f 14	*Dermatophagoides farinae*	37.5	42.9	Weak evidence
	282.4	UWT50543.1	Der f 14	*Dermatophagoides farinae*	37.5	41.2	Weak evidence
Glyceraldehyde 3-phosphate dehydrogenase	19.7	QBI57130.1	Per a 13	*Periplaneta americana*	90	100	Strong evidence
	26.9	QBA55500.1	Per a 13	*Periplaneta americana*	90	100	Strong evidence
	35.8	APD16997.1	Per a 13	*Periplaneta americana*	90	100	Strong evidence
Myosin heavy chain	216.8	AYC12378.1	Der f 11	*Dermatophagoides farinae*	52.5	–	Strong evidence

### Quantification of allergenic proteins

**Figure 2:**
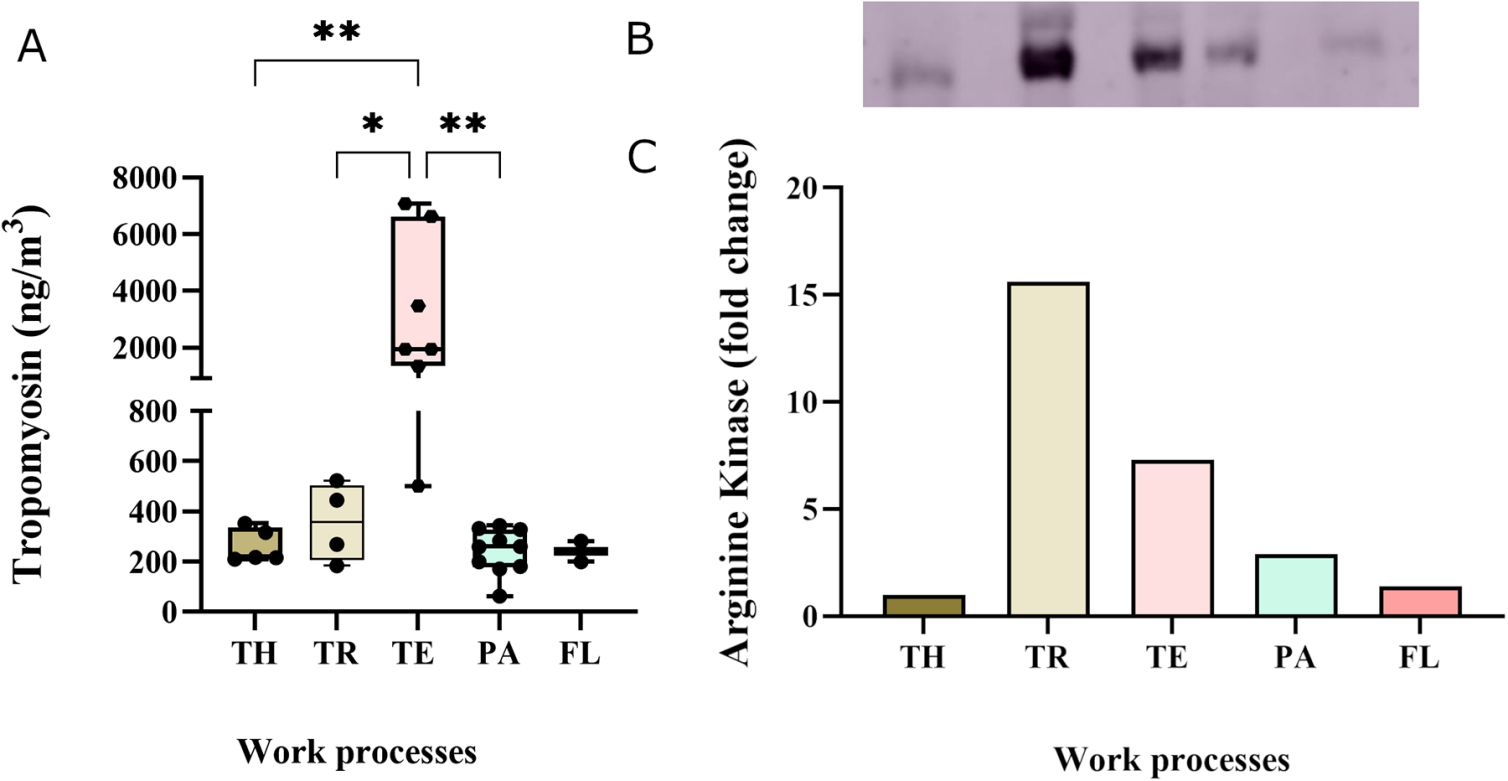
Exposure levels of allergenic proteins in personal aerosol samples across the five work processes: TH-thawers, TR-truck drivers, TE-technicians, PA-packers, and FL- flour production workers. **(A)** Tropomyosin levels TH (*n* = 6), TR (*n* = 4), TE (*n* = 7), PA (*n* = 11), and FL (*n* = 2). Group comparisons were done using one-way ANOVA, followed by Tukey's test. **(B)** Semi-quantitative analysis of arginine kinase levels by western blot, with bands representing samples pooled from each work process. **(C)** Graph showing the foldchange in arginine kinase concentration in relation to the thawing process.

**Figure 3:**
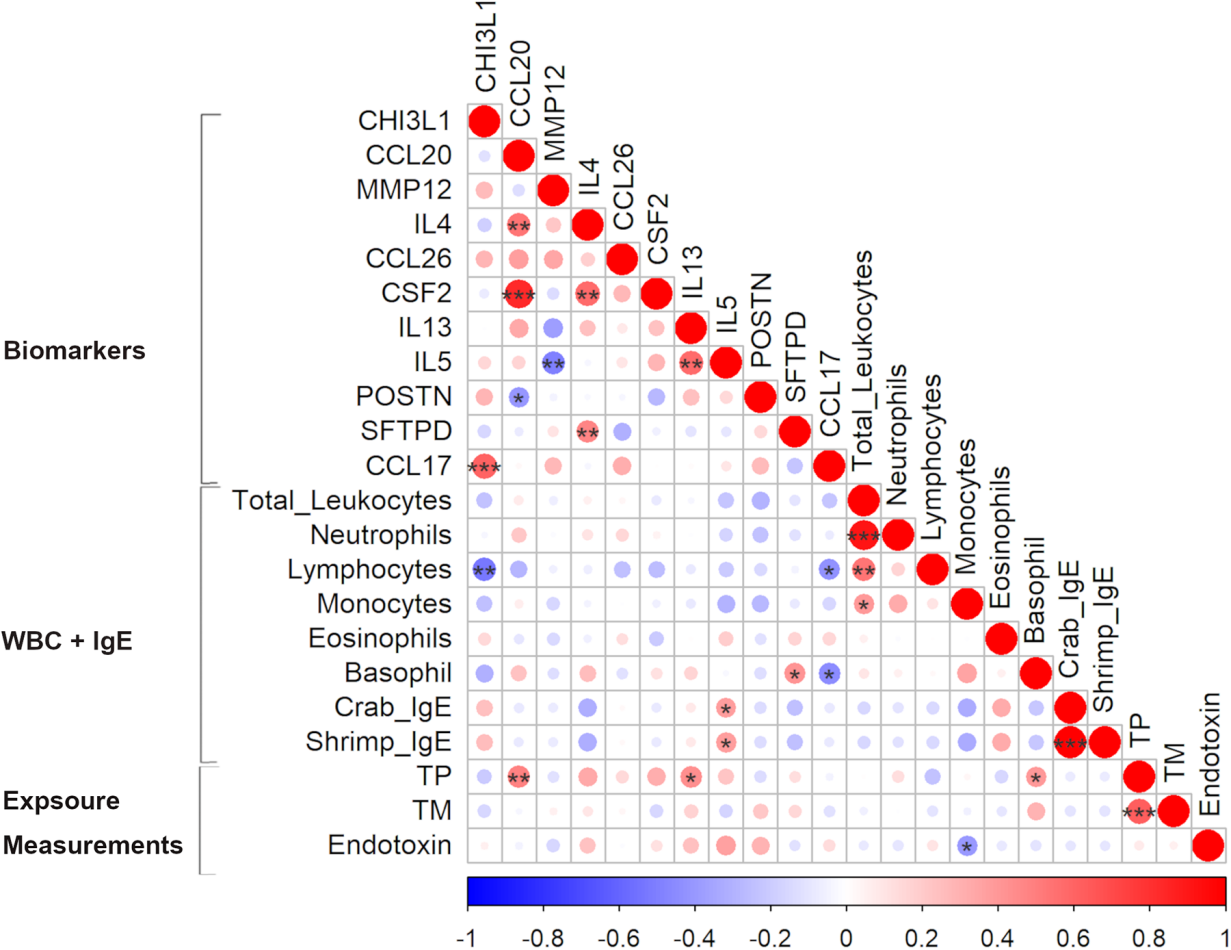
Pearson correlation matrix of biomarkers levels, white blood cell (WBC) count and exposure measurements from personal air samples (TP - total protein, TM—tropomyosin, and endotoxin) in exposed workers. Positive and negative correlations are indicated in red and blue, respectively. The colour intensity and size of the circles represent the strength of the correlations: larger and darker shades indicate a stronger correlation. *p < 0.05, **p < 0.01, ***p < 0.001.

The major allergen in shrimp, tropomyosin, was detected in personal samples in all work processes, with a median exposure level of 300 ng/m^3^ with a range of 62–7,070 ng/m^3^ (Figure 2A). The highest median tropomyosin level (1,928 ng/m^3^), measured among the technicians, was significantly higher than among thawers, truck drivers, and packers. The lowest median tropomyosin level was measured in the flour production (240 ng/m^3^) (Figure 2A). The tropomyosin levels were moderately correlated with the measured total protein levels (*r* = 0.63, *p* < 0.001) (Figure 3). In addition to tropomyosin, levels of arginine kinase, a known shrimp allergen, were semi-quantified by western blot. The analyses identified a truncated form of arginine kinase (∼30 kDa) in all five work processes (Figure 2B). The observed truncation or partial degradation of the protein was likely due to the industrial process itself or the post-sampling treatments, resulting in a protein smaller than the estimated molecular weight of arginine kinase (39–45 kDa). The result demonstrated that arginine kinase was present in all work processes. The exposure level of arginine kinase was higher among truck drivers and technicians, showing a 15-fold and 7-fold increase compared to the thawing, respectively (Figure 2C, Supplementary Figure S1).

### Protease activity

Protease activity was detected in 25% of the aerosol samples, predominantly among the thawers and truck drivers. While the observed band sizes were inconsistent with trypsin (Supplementary Figure S2A), one of the detected proteases was verified to be a metalloprotease (Supplementary Figure S2B,C).

### Correlations between exposure measures, biomarker levels and WBC among exposed workers

Correlation analysis showed that some exposure levels were correlated with a few of the biomarker levels. While tropomyosin levels did not correlate with any of the measured biomarkers, total protein was significantly correlated with CCL20 (*r* = 0.48, *p* = 0.01), IL13 (*r* = 0.44, *p* = 0.02) and basophil count (*r* = 0.39, *p* = 0.04) (Figure 3). In addition to the analysed protein levels, endotoxin levels are commonly measured to assess bioaerosol exposure in seafood industries. The measured endotoxin levels, ranging from LOD to 98 EU/m^3^, were included in the correlation analysis (see text footnote 1). A negative correlation between the measured endotoxin and monocyte count (*r* = −0.4, *p* = 0.04) was found and a positive borderline significant correlation with IL5 (*r* = 0.36, *p* = 0.05). Furthermore, the levels of crab IgE and shrimp IgE were strongly correlated (*r* = 0.99, *p* < 0.001) (Figure 3).

## Discussion

This study provides insight into the complex interaction between respiratory symptoms, biomarker levels in workers' blood, exposure to allergenic proteins, and irritants. The study showed that exposed workers reported more respiratory symptoms than controls. Particularly, acute upper respiratory symptoms and chronic lower respiratory symptoms were significantly prevalent among exposed workers compared to controls. In addition, exposed workers were four to five times more likely to report acute upper respiratory symptoms and chronic lower respiratory symptoms than controls. Similarly, workers in the shrimp, fish and crab industry frequently report symptoms of wheezing, shortness of breath and prolonged cough (8, 29, 46). In the present study, 22.9% of the exposed workers reported that they were diagnosed with asthma, and 11.4% of these workers were diagnosed with asthma as adults. This finding is comparable with a recent study by Laustsen and colleagues that reported 22.9% asthma incidents with 5.2% probable occupational asthma in the crab and shrimp industry (47). Occupational asthma is more frequently associated with workers in the shellfish processing industry than with the bony fish industry. Among the shellfish processing workers, the highest prevalence of occupational asthma (36%) has been reported in prawn processing plants (2). In the present study, 26.2% of the exposed workers reported having an allergy. However, most were related to pollen or grass, except for 2.9%, which reported shrimp allergy. Furthermore, 11% of the exposed workers exhibited elevated IgE levels against shrimp and crab in a specific allergy test. Furthermore, the IgE levels of shrimp and crab were strongly correlated, underscoring their cross-reactivity and potential sensitisation among the same workers (16, 48). Similarly, previous studies on shrimp processing workers in Greenland and Norway reported 10.1% and 20.3% of the workers, respectively, had elevated IgE levels against shrimp (8, 47). The improvement in the reported frequency of sensitisation in our study and the study by Laustsen et al. can likely be attributed to the continuous effort by companies to reduce exposure. In addition, individuals diagnosed with these allergies often seek alternative employment, resulting in a healthy worker effect, which may potentially lead to an underestimation of the exposure-related effects on symptom prevalence.

In addition to the respiratory symptoms reported in the present study, differential blood counts showed that 7.1% of the exposed workers exhibited higher levels of total leukocytes, lymphocytes, monocytes, and eosinophils.

To our knowledge, this study is the first to analyse the levels of allergy and asthma biomarkers in exposed workers in this industry, comparing them to controls, thereby offering new insight into the immune response in exposed workers. Our study showed that exposed workers exhibited significantly higher levels of IL4 relative to controls. This finding indicates an enhanced allergenic response, which could be caused by exposure to allergenic proteins such as tropomyosin (31, 32, 49). Additionally, the data shows substantially higher levels of CCL20, CSF2, and MMP12 among exposed workers than controls. These biomarkers are known to be involved in inflammation, asthma, and allergy pathways, further supporting the notion that exposure to allergenic proteins and irritants in shrimp processing plants may exacerbate allergenic and inflammatory reactions (35, 37, 50). CCL17 levels were lower among exposed workers and workers with allergies, and those who reported acute upper respiratory symptoms while reported chronic lower respiratory symptoms were associated with higher CCL17 levels. Similarly, increased CCL17 levels have been previously reported in the serum of patients with allergic asthma (51), while CCL17 levels were unaltered in patients with allergic rhinitis. The observed increase in CCL17 levels is likely related to the persistent inflammation that characterises chronic respiratory conditions, where elevated CCL17 levels help recruit immune cells to sustain and modulate the ongoing inflammatory response (35) Thus, while CCL17 is important in allergic inflammation, its expression in plasma may be affected by disease stage and severity. Altogether, this indicates a need for more research to evaluate the suitability of CCL17 as a biomarker of acute vs. chronic occupational respiratory symptoms.

Overall, the observed levels of these biomarkers and the increased WBC indicate that exposed workers have a higher incidence of immunological and allergic responses. Furthermore, exposed workers had significantly elevated levels of SFTPD and CHI3L1 following a work shift, showing an acute inflammatory response to the occupational exposure. These two markers have been implicated in the first line of defence and inflammatory response and tissue remodelling (39, 43, 52). Thus, the significant elevation of SFTPD and CHI3L1 levels post-shift might suggest the initiation of allergenic rhinitis following bioaerosol exposures, which may be linked to the reported acute upper respiratory symptoms (41).

While the variation in biomarker levels provides valuable insights into the physiological responses of workers, it is equally important to examine the types and levels of exposure in this work environment. Assessing these, along with the biomarkers, can help establish a potential link to respiratory symptoms. Total protein measurements are often used in the seafood industry. In the present study, up to 66.2 µg/m^3^ was measured in the personal air samples and the highest levels were measured in the cooking and peeling department. This finding aligns with another study conducted in a shrimp processing plant where they measured 50 µg/m^3^ (47). Many of these processing plants still use thermal processing, which may increase protein aerosolisation and allergenicity by altering the protein structure and creating new epitopes (13). Earlier studies in crab processing plants have reported as much as 6 mg/m^3^ protein (53). The total protein levels in the present study were found to be significantly correlated with the level of CCL20, IL13 and basophil count in exposed workers. The correlation suggests a collective role of these markers in the immune response to potential allergenic proteins. However, it also underscores that total protein levels provide limited information as not all proteins provoke sensitisation upon inhalation (54). Therefore, it is essential to investigate also the composition of allergenic proteins in the air. Previous studies have reported total allergen levels, based on reactivity to patient sera, of 6.3 μg/m^3^ in the shrimp processing industry and 75 μg/m^3^ in the fish industry (8, 53). However, these studies do not identify the causative allergen. In this study, we measured the levels of the major shrimp allergen tropomyosin in the aerosol samples and very high levels were observed, ranging from 62 to 7,070 ng/m^3^. The highest tropomyosin level, 7,070 ng/m^3^, was measured among the technicians in the cooking and peeling department. Moreover, all other work processes also exhibited relatively high tropomyosin levels compared to the historically reported 375 ng/m^3^ in shrimp processing plants (7). In addition, the peak tropomyosin level recorded in this study also surpassed the previously reported levels (5,133 ng/m^3^) in prawn processing plants (5). These levels significantly exceed what has been reported in the crab processing industry, which ranged from 0.15 to 138 ng/m^3^ (55). While we showed high levels of tropomyosin in the aerosol, the levels of tropomyosin were only moderately correlated to the total protein, indicating that additional factors contribute to the reported respiratory symptoms among exposed workers. Characterisation of the composition of the allergenic proteins in the aerosol showed that the allergenic burden in the shrimp processing industry is complex, and both other known allergens such as arginine kinase, sarcoplasmic calcium-binding protein, troponin C, triosephosphate isomerase, myosin light chain and hemocyanin, as well as potential novel allergens may contribute to the observed respiratory symptoms among exposed workers. While we reported very high levels of the major known allergen tropomyosin, the contribution of other allergens is also of interest. Although methods to quantify the other identified allergies are not yet available, we conducted a semi-quantitative analysis of arginine kinase. The results showed arginine kinase was present in all work processes, with the higher levels observed among truck drivers and technicians. Previously, Abdel Rahman and colleagues have reported an arginine kinase level of 420 ng/m^3^ in the shrimp processing industry using mass spectrometric analysis (7).

In addition to allergenic proteins, proteases such as trypsin may induce airway inflammation and exacerbate allergic responses through disruption of epithelial barriers and release of inflammatory mediators (23, 25, 27). In the present study, proteases were identified in personal samples collected primarily from the thawing department. While trypsin was not detected in any of the samples, metalloproteases were observed in one sample. Exposure to these proteases can stimulate the airways through the attraction of eosinophils and neutrophils, a reaction that resembles the response to allergens (22). Trypsin is predominantly located in the digestive system and is believed to be released during the gutting processes in seafood processing; thus, its absence in the shrimp industry is unsurprising. In shrimp processing, there is deveining rather than gutting, and the small size of the shrimp further contributes to the absence of trypsin. In addition, exposure to chitin has also been associated with pulmonary inflammation and asthma (19–21). High exposure levels of chitin are expected in the flour production department, where the shell is dried and ground, and could thus contribute to the reported health effects among exposed workers. Finally, while allergens are a major sensitising factor in the seafood industry, other factors, such as chemicals, endotoxins and microbes, may also contribute to respiratory ailments (8, 56). The endotoxin levels we measured in these shrimp processing plants were largely below the recommended occupational exposure levels of 90 EU/m^3^ (57). Despite these low endotoxin levels, significant negative correlation between the endotoxin levels and monocyte count was observed, indicating that the presence of endotoxin could also contribute to the immune response observed among exposed workers.

### Strengths and limitations

This study evaluated the allergenic exposure in shrimp processing plants, linking the exposure to known biomarkers of allergy and asthma. The comprehensive mapping of the level and composition of allergenic proteins in the work environment enhanced and strengthened the study. While it provides novel insight into occupational exposure and respiratory health issues among shrimp processing workers, its cross-sectional design and the relatively low sample size restrict its generalisation and limit the establishment of causality, further warranting the need for larger studies to strengthen and corroborate the findings.

In this study, respiratory symptoms were assessed as self-reported symptoms through questionnaires. Self-reporting may cause reporting bias, and additional clinical evaluations of respiratory symptoms would strengthen the study. Furthermore, it is important to note that the healthy worker effect is common in industries with a high prevalence of allergic symptoms, as workers experiencing health problems often leave the workforce or switch professions. This may result in an underestimation of the health impacts of occupational exposures (58, 59).

Finally, the study was limited by methodological challenges due to the lack of a complete sequencing of *Pandalus borealis* in the reference database. Consequently, protein identification was done based on homology with other species within the infraorder Caridea. This resulted in low coverage for some proteins with low homology, restricting the interpretation and highlighting the need for additional studies to verify these findings. Moreover, protocols for extracting proteins from air filter samples are not standardised. Protocol selection may potentially introduce bias in the representative composition of proteins in the samples.

## Conclusion

This study reports a high prevalence of respiratory symptoms and respiratory sensitisation, accompanied by elevated levels of allergy and asthma biomarkers in exposed shrimp processing workers. It provides novel insight into the level and composition of allergenic exposure in the shrimp processing industry, demonstrating alarmingly high tropomyosin levels in specific work tasks. Therefore, developing suitable monitoring tools, such as high throughput standardised assays for measurement and monitoring of specific allergens, could contribute to identifying potential health-impairing exposures and help to evaluate exposure-reducing measures. Altogether, these findings underscore the importance of mitigating occupational exposure to allergenic proteins to reduce the risk of allergies and asthma among workers. This can be achieved by introducing targeted preventative initiatives in high-exposure processes, such as implementing engineering controls (including effective ventilation systems) or using personal protective equipment.

## Data Availability

The proteomics data are available in the publicly accessible Proteomic Identification Database (PRIDE) partner repository with the dataset identifier PXD061442. Additional original data presented in this study are included in the supplementary materials. The questionnaire data presented in this article are not readily available due to ethical concerns. Requests to access these datasets should be directed to the corresponding author.

## Footnotes

^1^Zegeye FD, Straumfors A, Lei P, Graff P, Erdem JS, Afanou AK. Microbial exposure and diversity in Norwegian shrimp processing plants. *J Occup Environ Hyg*. (2025).
