# Supplementary material for: Respiratory symptoms, sensitisation and occupational exposure in the shrimp processing industry
Source: Front Allergy. 2025 Mar 20;6:1520576. doi: 10.3389/falgy.2025.1520576 (PMC11967198; doi:10.3389/falgy.2025.1520576)
Supplement: Supplementary file 1 [file Supplementaryfile1.zip › Supplementary Tables.docx]

Supplementary material for respiratory symptoms, sensitisation and occupational exposure in the shrimp processing industry

# Supplementary 1: Additional Results

**Table S1: Prevalence of reported asthma and allergy**

|  | **Exposed workers** | **Control (Unexposed)** |
| --- | --- | --- |
|  | N = 35 | N = 21 |
|  |  |  |
| **Asthma diagnosis by general practitioner (%)** ^a^ | **22.9** | **4.8** |
| Asthma attack in the past year (%) | 2.8 | 0 |
| Asthma medication use (%) | 5.7 | 0 |
| Asthma diagnosis as adult (%) | 11.4 | 4.7 |
| **Allergy (%)** ^b^ | **26.2** | **42.9** |
| Pollen allergy (%) | 5.8 | 19 |
| Grass allergy (%) | 5.8 | 14.3 |
| Cat and dog allergy (%) | 2.9 | 0 |
| Shrimp (%) | 2.9 | 0 |
| Dust allergy (%) | 0 | 4.8 |
| Wasp (%) | 0 | 4.8 |
| Unspecified | 8.8 | 0 |

^a^ Fisher test was used, no significant difference was observed. ^b^ Pearson chi-square test was used, no significant difference was observed.

**Table S2: Specific IgE test and differential count among exposed workers**

|  | **Exposed production workers** |
| --- | --- |
|  | N = 35 |
|  |  |
| Crab IgE > 0.35 kU/L | 11 % |
| Shrimp IgE > 0.35 kU/L | 11 % |
| Salmon IgE > 0.35 kU/L | 3.7 % |
| Total Leukocyte > 10 ×10^9^/L | 7.1 % |
| Neutrophils > 7.3 ×10^9^/L | 3.5 % |
| Lymphocytes > 3.3 ×10^9^/L | 7.1 % |
| Monocytes > 0.8 ×10^9^/L | 7.1 % |
| Eosinophils > 0.4 ×10^9^/L | 7.1 % |
| Basophils > 0.2 ×10^9^/L | 0 % |

**Table S3. Novel allergens identified with AllerCatPro2**

|  |  |  |  |  | **Shrimp species** | **Predicted most similar allergen** | | **Similarity to allergen and resulting predicted evidence for allergenicity** | | |
| --- | --- | --- | --- | --- | --- | --- | --- | --- | --- | --- |
| **Protein name** | **MW [kDa]** | **Coverage [%]** | **# Peptides** | **Accession #** | **Previously the allergen identified in** | **Protein allergen** | **Other species with similar allergen** | **% identity, linear 80 aa window** | **% identity, 3D epitope** | **Result** |
| Alpha-tubulin | 39.4 | 12 | 3 | QBS13807.1 | *Macrobrachium nipponense* | Lep d 33 | *Lepidoglyphus destructor* | 98.8 | 100 | strong evidence |
| Chitinase | 54.2 | 7 | 3 | AFC60658.1 | *Pandalus japonicus* | Bla g 12 | *Blattella germanica* | 70 | 84.6 | weak evidence |
| Heat shock protein 70 | 71.2 | 8 | 5 | ACL30943.1 | *Rimicaris exoculata* | Tyr p 28 | *Tyrophagus putrescentiae* | 95 | 100 | strong evidence |
|  | 66.3 | 7 | 4 | ACL52279.1 | *Rimicaris exoculata* | Der p 28 | *Dermatophagoides pteronyssinus* | 92.5 | 100 | strong evidence |
|  | 71.5 | 8 | 5 | ADN78256.1 | *Palaemon carinicauda* | Tyr p 28 | *Tyrophagus putrescentiae* | 98.8 | 100 | strong evidence |
| Pyruvate kinase | 57.5 | 17 | 7 | ALK82311.1 | *Macrobrachium nipponense* | Sal s 9 | *Salmo salar* | 81.2 | - | strong evidence |
| Troponin T | 45.7 | 26 | 14 | AQV08184.2 | *Palaemon carinicauda* | Pon l 7 | *Astacus leptodactylus* | 93.8 | - | strong evidence |
| Vitellogenin | 283.1 | 36 | 77 | QCS40650.1 | *Pandalus platyceros* | Der f 14 | *Dermatophagoides farinae* | 37.2 | 40 | weak evidence |
|  | 284.6 | 19 | 41 | AHD26978.1 | *Pandalus japonicus* | Der f 14 | *Dermatophagoides farinae* | 35 | 37.5 | weak evidence |
|  | 283 | 35 | 82 | ACU51164.1 | *Pandalus japonicus* | Der f 14 | *Dermatophagoides farinae* | 33.8 | 50 | weak evidence |
|  | 283.4 | 37 | 82 | BAD11098.1 | *Pandalus hypsinotus* | Der f 14 | *Dermatophagoides farinae* | 35 | 50 | weak evidence |
|  | 287.6 | 2 | 5 | AFM82474.1 | *Palaemon carinicauda* | Der f 14 | *Dermatophagoides farinae* | 37.5 | 42.9 | weak evidence |
|  | 282.4 | 1 | 3 | UWT50543.1 | *Hippolyte inermis* | Der f 14 | *Dermatophagoides farinae* | 37.5 | 41.2 | weak evidence |
| Glyceraldehyde 3-phosphate dehydrogenase | 19.7 | 33 | 5 | QBI57130.1 | *Pandalus borealis* | Per a 13 | *Periplaneta americana* | 90 | 100 | strong evidence |
|  | 26.9 | 20 | 5 | QBA55500.1 | *Nautilocaris saintlaurentae* | Per a 13 | *Periplaneta americana* | 90 | 100 | strong evidence |
|  | 35.8 | 22 | 7 | APD16997.1 | *Macrobrachium olfersii* | Per a 13 | *Periplaneta americana* | 90 | 100 | strong evidence |
| Myosin heavy chain | 216.8 | 29 | 65 | AYC12378.1 | *Palaemon carinicauda* | Der f 11 | *Dermatophagoides farinae* | 52.5 | - | strong evidence |
