# Supplementary material for: Respiratory symptoms, sensitisation and occupational exposure in the shrimp processing industry
Source: Front Allergy. 2025 Mar 20;6:1520576. doi: 10.3389/falgy.2025.1520576 (PMC11967198; doi:10.3389/falgy.2025.1520576)
Supplement: Supplementary file 1 [file Supplementaryfile1.zip › Supplementary Data 1.pdf]

Supplementary material for respiratory symptoms, sensitisation and occupational exposure in the shrimp processing industry

Supplementary 2: Questionnaire used in the study:

EXPOSURE TO BIOAEROSOLS IN THE SEAFOOD INDUSRTY

ID-code ☐☐ Date (dd.mm.yyyy) ☐☐☐

QUESTIONNAIRE ON WORK ENVIRONMENT AND HEALTH IN THE PRAWN INDUSTRY

To answer the questions, please tick off the most appropriate box. If you are uncertain what to answer, choose «No». For all questions, try to give the best possible answer. Take your time! Your answers are very important for the results of this study.

Thank you for your cooperation!

I. PERSONAL INFORMATION

-

Male ☐ Female☐ Other ☐

Age _______________ *years*

Height _________________ *cm* Weight _____________________ *kg*

II. SMOKING/SNUFF

2.1.Do you smoke daily? ☐Yes ☐ No

- If Yes:

How many years have you been smoking? Number of years: __________

- How many cigarettes do you smoke a day? Number of cigarettes per day: _______

2.2.Do you use snuff? ☐Yes ☐ No

- If Yes:

How much do you use a day? Number of snuff units: ___________

Strength: ________________________

2.3. Do you smoke e-cigarettes? ☐Yes ☐ No

- If Yes:

What type of e-cigarettes are you using?
Without nicotine ☐
With nicotine ☐ Strength: ________________________
Without nicotine, with flavoring ☐
With nicotine, without flavoring ☐ Strength: ________________________

Answer if you do not currently smoke:

2.4.Have you smoked on a daily basis earlier in life? ☐Yes ☐ No

- If Yes:
  - Did you quit smoking more than 1 year ago? ☐Yes ☐ No
  - How many years did you smoke in total? Number of years: _________

How many cigarettes, on average, did you smoke a day? Cigarettes per day: ________

III. ALLERGIES

3.1. Do you have any allergies, such as pollen, animal hair, etc.? ☐Yes ☐ No

- If Yes, what are you allergic against: ________________________________
- If Yes, which symptoms do you show?
  a. Eye related symptoms such as itching, tearing, etc. ☐Yes ☐ No
  b. Nose related symptoms such as sneezing, obstructed or running nose ☐Yes ☐ No
  c. Asthma ☐Yes ☐ No
  d. Skin related symptoms such as eczema, itching, rash, etc. ☐Yes ☐ No

  Other: ____

IV. ASTHMA

4.1. Have you been diagnosed with asthma by a doctor? ☐Yes ☐ No

- If Yes,
  a. As child or adolescent ☐Yes ☐ No
  b. As adult ☐Yes ☐ No
  c. I still have asthma ☐Yes ☐ No

4.2. Have you had an asthma attack during the past 12 months? ☐Yes ☐ No

4.3. Do you use any asthma medication (inhaler, aerosols, pills)? ☐Yes ☐ No

---

V. RESPIRATORY COMPLAINTS

5.1. Which of the following describes your breathing best?

a. I never/hardly ever have trouble breathing. ☐Yes ☐ No

b. I regularly have trouble breathing. ☐Yes ☐ No

c. I always have trouble breathing. ☐Yes ☐ No

5.2. Have you suffered from flu-like symptoms in the past 12 months? ☐Yes ☐ No

- e.g. fever/chills that lasted more than two days.

5.3. Have you experienced shortness of breath, without making any physical ☐Yes ☐ No

- effort, during the past 12 months?

5.4. Have you had wheezing or peeping noise in your chest in the past 12 months? ☐Yes ☐ No

5.5. Do you usually cough when you wake up in the morning? ☐Yes ☐ No

5.6. Do you usually produce phlegm when you wake up? ☐Yes ☐ No

5.7. In sum, do you cough daily for three or more months during a year? ☐Yes ☐ No

---

VI. SKIN

6.1. Have you ever had hand-eczema? ☐Yes ☐ No

6.2. Have you ever had eczema on your wrists or forearms? ☐Yes ☐ No

- (except inside of elbows.)
- **If you answered «No» to questions 6.1. and 6.2., move on to question 6.7. If you answered «Yes», please proceed with question 6.3.**

6.3. Have you noticed that contact with certain materials, substances, chemicals or other during your workday worsens your eczema?

a. Eczema on hand ☐Yes ☐No

b. Eczema on wrist/forearm ☐Yes ☐No

- If Yes, what chemical?

____

6.4. Have you noticed that contact with certain materials, substances, chemicals or other <u>during your leisure time</u> worsens your eczema?

a. Eczema on hand ☐Yes ☐No

b. Eczema on wrist/forearm ☐Yes ☐No

If Yes, what chemical? ____

6.5. Does you eczema get any better during periods when you are away from work? (e.g. weekends, holidays or other extended absence.)

c. Eczema on hand ☐Yes ☐No

d. Eczema on wrist/forearm ☐Yes ☐No

6.6. Did you have eczema as a child? ☐Yes ☐ No ☐Don't know

Were you diagnosed by a doctor? ☐Yes ☐ No

At what age were you diagnosed? _______________ (years)

VIII. ILLNESS AND MEDICATION

8.1. Have you been diagnosed with emphysema, COPD, heart conditions or a similarly serious health condition? ☐Yes ☐ No

Please indicate which illness and if you are still suffering: ________________________________

____

8.2. Do you use prescription medications? ☐Yes ☐ No

Please indicate which medications you use: ____

____

-
-

QUESTIONS ABOUT WORK-RELATED SYMPTOMS AND HEALTH COMPLAINTS DURING THE PAST WEEK. (While at work and during the two first hours after you left for the day.)

	Yes, often	Yes, sometimes	No
Eczema on hands	☐	☐	☐
Dry skin on hands	☐	☐	☐
Runny, itchy, sore or red eyes	☐	☐	☐
Fever	☐	☐	☐
Sneezing	☐	☐	☐
Stuffy nose	☐	☐	☐
Runny nose	☐	☐	☐
Sore throat	☐	☐	☐
Coughing with phlegm	☐	☐	☐
Coughing with sputum	☐	☐	☐
Wheezing	☐	☐	☐
Congested chest	☐	☐	☐

Headache	☐	☐	☐
Fatigue or weakness	☐	☐	☐
Vomiting or nausea	☐	☐	☐
Diarrhoea	☐	☐	☐
Eczema/dry red skin in the face	☐	☐	☐
