# Supplementary material for: Respiratory symptoms, sensitisation and occupational exposure in the shrimp processing industry
Source: Front Allergy. 2025 Mar 20;6:1520576. doi: 10.3389/falgy.2025.1520576 (PMC11967198; doi:10.3389/falgy.2025.1520576)
Supplement: Supplementary file 1 [file Supplementaryfile1.zip › Supplementary Figures.PDF]

Supplementary material for respiratory symptoms, sensitisation and occupational exposure in the shrimp processing industry

Supplementary 1: Additional Results

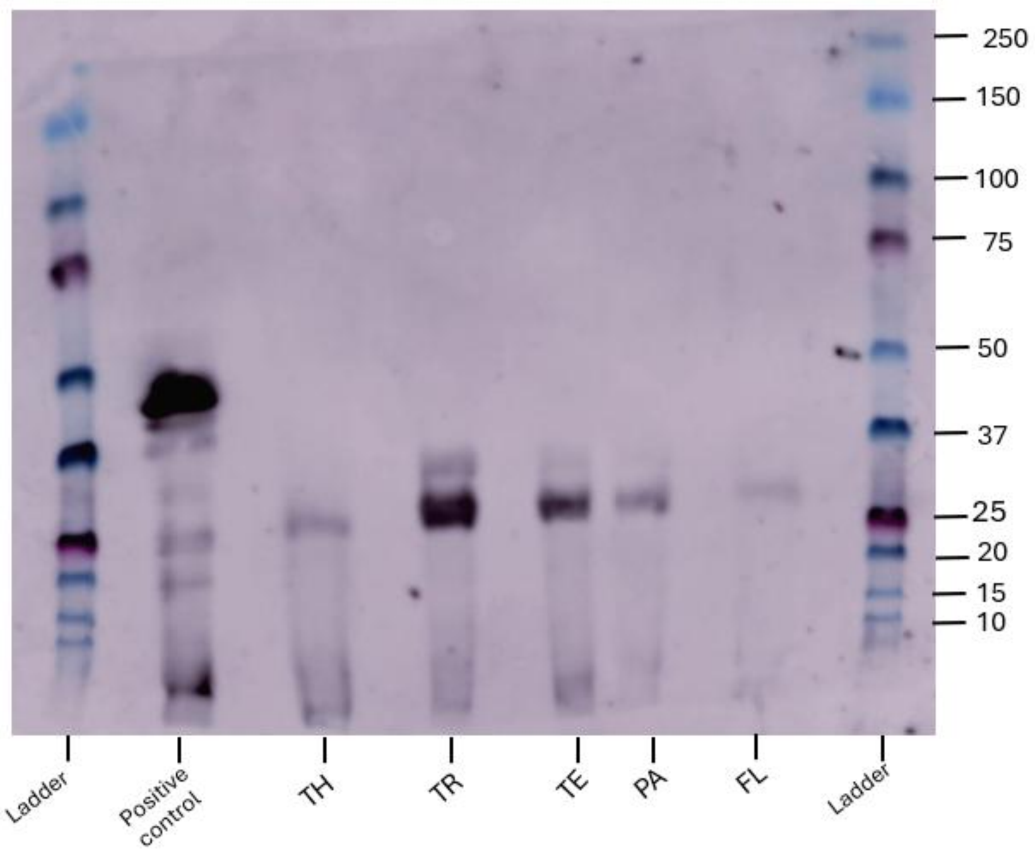

Fig. S1: Western blot analysis using specific anti-arginine kinase antibody performed on pooled filter extract from personal samples across five work groups (TH-thawers, TR-truck drivers, TE-technicians, PA-packers, and FL- flour production workers). Protein extracted from shrimp was used as a positive control.

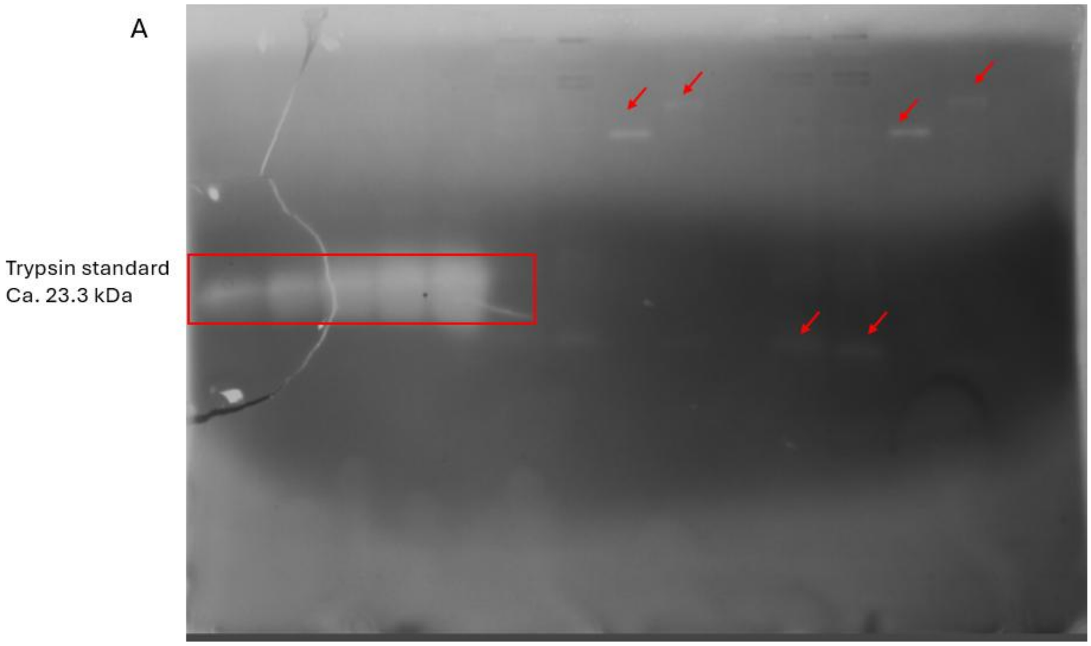

EDTA inhibition experiment

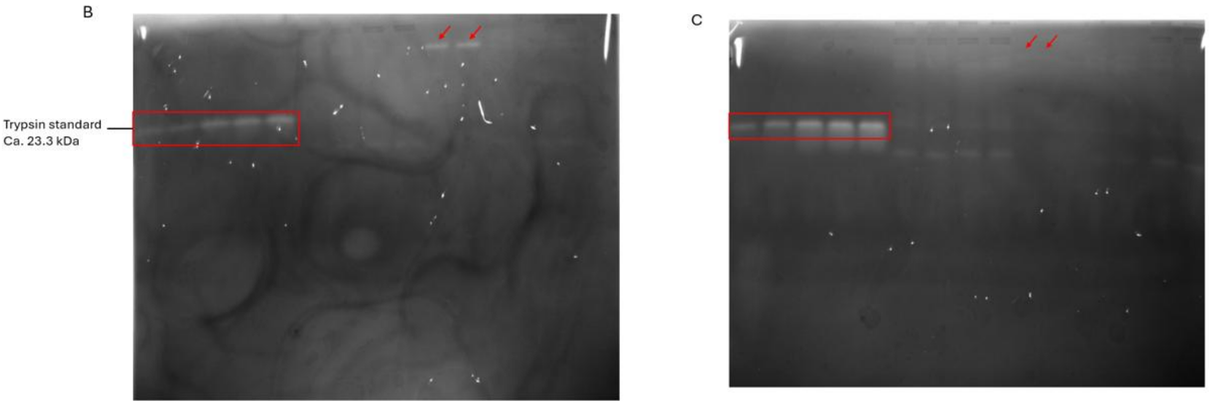

Fig. S2: Zymography assay gels a) Samples showing proteolytic activity inconsistent with trypsin. b) Gel without EDTA incubation showing a specific bond c) Gel incubated in EDTA showing the inhibition of the same sample.
